# Novel Tools to Assess Muscle Sarcopenic Process in ICU Patients: Are They Worthwhile?

**DOI:** 10.3390/jcm12103473

**Published:** 2023-05-15

**Authors:** Sergio Ruiz-Santana, Carmen Rosa Hernández-Socorro

**Affiliations:** 1 ICU, Hospital Universitario de Gran Canaria Dr. Negrín, 35010 Las Palmas de Gran Canaria, Spain; 2 Department of Medical and Surgical Sciences, University of Las Palmas de Gran Canaria, 35016 Las Palmas de Gran Canaria, Spain; 3 Radiology Department, Hospital Universitario de Gran Canaria Dr. Negrín, 35010 Las Palmas de Gran Canaria, Spain; chersoc@gobiernodecanarias.org; 4 Department of Clinical Sciences, University of Las Palmas de Gran Canaria, 35016 Las Palmas de Gran Canaria, Spain

Critical illness induces hypercatabolic response with severe loss of lean body mass, this being a key symptom in patients with prolonged ICU stay and is associated with acquired muscle weakness, long-term mechanical ventilation, fatigue, delayed recovery, and poor quality of life after ICU stay [1,2]. It has been recently estimated that these patients lose nearly 2% of skeletal muscle per day during the first week of ICU admission [3].The sarcopenia stage in ICU patients is also eventually characterized by low muscle mass in addition to low muscle strength or physical performance. It has been observed that there is no direct relationship between muscle mass and muscle function, and it should be noted that muscle function tests during critical illness are difficult to perform and evaluate, such as the MRC (“Medical Research Council”) scale, which requires the patient’s cooperation, i.e., that he/she is awake [2]. In sedated patients, there are few studies with contradictory results on the value of techniques such as neuromuscular electrical stimulation usually combined with usual care, exercise, or protein supplementation [4,5]. Histopathological analysis may be necessary to clarify the underlying pathophysiological mechanisms but cannot be part of clinical practice [6]. Hence, noninvasive, bedside imaging techniques are more attractive in the study of muscle wasting.

Different imaging techniques at the tissue level are very useful for the study of body composition and, therefore, for sarcopenia assessment. Magnetic Resonance Imaging (MRI) and abdominal Computed Tomography (CT) have been used in the study of body composition and to establish the predictive value of sarcopenia and metabolic alterations [7,8,9,10,11].

Repeated abdominal and femoral CT scans have been performed to manage temporary muscle loss and to assess the impact of nutrition on muscle and adipose tissue during critical illness, but CT and MRI are not techniques that can be repeated in clinical practice. CT is a safe measure, which generates ionizing radiation and should not be repeated, and is of little use in the ICU [8,9,10]. MRI is a highly safe measure for assessing nutritional status, but is costly and specialized, and is not useful in daily practice in the ICU [7,11].

Ultrasound (US) is a portable, safe, reliable, and inexpensive bedside technique that has proven to be very useful in the diagnosis of sarcopenia and in the management of critically ill patients and allows monitoring sarcopenia and long-term muscle quality in these patients, compared to the best-known techniques. Its usefulness in the study of visceral and subcutaneous adipose tissue at the abdominal level has been developed a long time ago, and isa promising technique, at present, in the assessment of musculoskeletal tissue [2,6]. The muscle US technique is not easy to perform since it has a long learning curve due to the multiple errors that can occur in its execution. If properly handled, it has high reliability in the study of muscle thickness and muscle cross-sectional area [12,13]. It has several disadvantages, which are as follows: lack of standardization of measurement techniques, results being affected by technical errors, lack of protocols, and anatomical variants. It produces more qualitative than quantitative results and its greatest difficulty is in interpreting or discerning the contours of the muscle and its adjacent structures. It is, therefore, a technique highly dependent on the operator’s skills [12,13].

The technical errors caused by the ultrasonographic technique are as follows: compressibility, selection of the right place to perform the study, the optimal position of the transducer, muscle relaxation or contraction, and hydration. Errors can be avoided by not squeezing the muscle and using a lot of gel, selecting a place to perform serial measurements, and placing the probe parallel or perpendicular to the muscle to be studied, with an insonation angle perpendicular to the longitudinal axis [2,12]. Some studies establish the reliability of US in muscle thickness and the cross-sectional area of the muscle and it is, therefore, useful as a marker of the presence of muscle disease and is very promising as a marker of evolution or regression of the same [12,13,14,15,16].Further studies are needed regarding the quantification of echogenicity to detect reduction or improvement in disease progression. 

Given these data, objective measures were needed to detect muscle involvement, i.e., biomarkers of “muscle wasting”. Therefore, we first applied several ultrasound techniques previously described and investigated in sports, physiology [17], chronic obstructive pulmonary disease, and elderly population studies [16,18], such as elastography (SWE), superb microvascular imaging (SMI), and contrast-enhanced quadriceps anterior rectus muscle ultrasound (CEUS), to accurately analyze muscle quantity and quality in critically ill patients [19]. Tissue Doppler imaging and SWE have also been studied in real-time diaphragmatic motion and qualitative assessment in ICU patients to assess muscle injury and weakness and differentiate patients who fail or pass a weaning trial [20,21]. Although it has been previously shown that SWE muscle analysis may provide new data about muscle quality during critical illness in twelve ICU patients and healthy controls in a French study [22], we rather investigated SWE in ICU patients with prolonged mechanical ventilation and with clinical suspicion of critical patient weakness [19].

We found that SWE showed, with an outstanding area under the ROC curve, that patients acquired relevant muscle stiffness due to fibrosis with important clinical consequences (Figure 1). 

We also performed, in our study, SMI for the first time in this type of patient; SMI is a new ultrasound software that allows detecting minor alterations in muscle microvascularization. When low muscle vascular flow is observed, it is a clear primary sign of muscle vitality and indicates that muscle recovery is feasible (Figure 2). 

In addition, we used CEUS to investigate muscle perfusion (Figure 3). 

Indeed, CEUS performed slightly worse than the other markers investigated. However, when used as a biomarker of SMI, its diagnostic ability increased markedly [19]. All of the above findings with these novel ultrasound tools are important because, for the first time, it has been shown that they can be used to assess the muscle wasting process in this cohort of ICU patients and, because of their relevance, should be incorporated into current clinical protocols for musculoskeletal ultrasound in ICU patients [19].

Once diagnosed with severe sarcopenia by the US, what can physicians do? Are there any changes in treatment that can improve these poor outcomes? Treatment of these patients should consist of recognition and prevention. The latter involves early treatment of conditions leading to multiorgan failure, such as sepsis and septic shock, control of excessive sedation, control of hyperglycemia, and limiting the use of corticosteroids and muscle blockers [2]. Likewise, neuromuscular electrical stimulation and early active mobilization of patients on mechanical ventilation are advised, as well as resistance exercises, through an interdisciplinary team, with physiotherapy and rehabilitation programs, although the suitability of most of these measures has also been questioned recently [4,23].

It is also of great interest to develop specific therapies aimed at halting or decreasing anabolic resistance and immobilization and attempting to increase nutritive muscle blood flow and microvascular blood flow. Among these therapies, in a pilot study, a short-term multimodal intervention using protein supplementation, electrical stimulation, and exercise appeared to be able to mitigate lower extremity muscle loss in ICU patients [5].

On the other hand, the use of the leucine metabolite beta-hydroxy beta-methyl butyrate (HMB), which appeared to be the most promising for attenuating muscle wasting in preliminary studies, failed to reduce muscle wasting when administered for 10 days versus placebo [24]. Studies on the use of fish oil supplements, rich in eicosapentaenoic acid and docosahexaenoic acid, which may help attenuate muscle strength loss and soreness with exercise, also appeared promising. However, these therapeutics have only recently been investigated in critically ill patients and, again, their usefulness in these patients remains to be defined [25].

In summary, imaging techniques should help link radiological changes with the clinical findings of patients during their stay in the ICU and their post-ICU functional status. US is a technique in growing development in muscle studies due to its good correlation with MRI and CT, being safe in the precision of the measurements, non-invasive, reliable, portable, and performed at the patient’s bedside [13,18]. By applying the US protocolized usual muscle measurements together with the recently described muscle novel tools, we are going to influence the diagnosis, prognostic, and therapeutic attitude in many critically ill patients. A reliable and more frequent diagnosis of sarcopenia with these techniques in ICU patients will result in different clinical management and ultimately better clinical outcomes. Further research is needed in this cutting-edge field. These novel US tools are worthwhile, indeed.

## Figures and Tables

**Figure 1:**
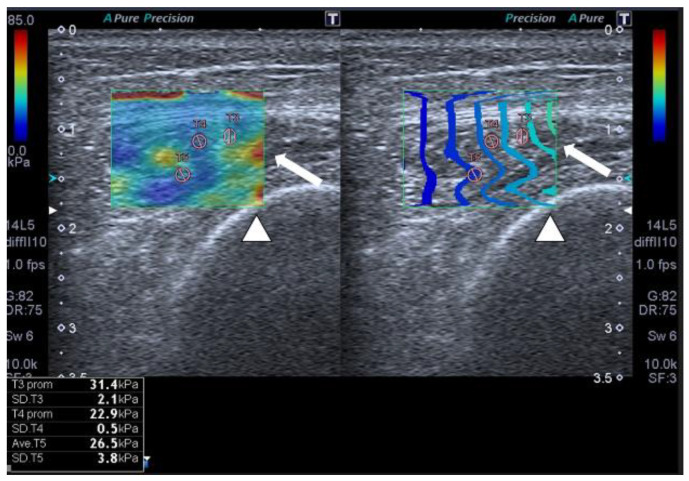
SWE shows muscle stiffness with several regions of interest (ROIs) in a 56-year-old patient with multiorgan failure. Transversal QRF muscle US scan (arrow), femur (arrowhead).

**Figure 2:**
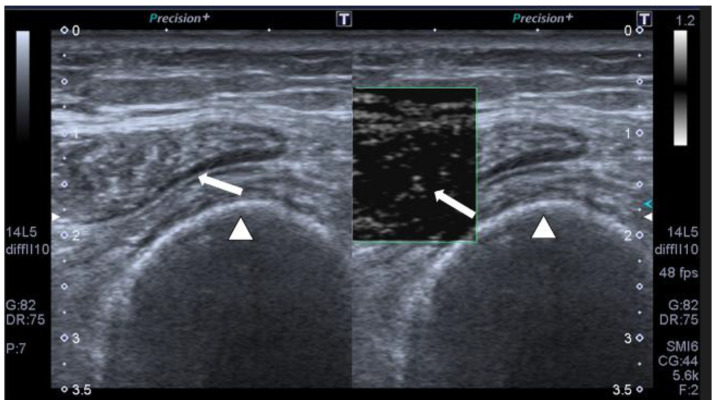
SMI monochromatic mode image shows minimal linear vessels in a 56-year-old patient with multiorgan failure on transverse muscle ultrasound QRF (arrow), femur (arrowhead).

**Figure 3:**
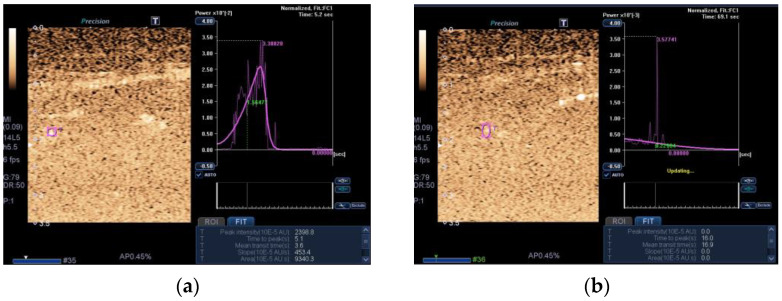
Transversal ultrasound of the QRF muscle after CEUS administration and time-intensity curve analysis in a 56-year-old patient with multiorgan failure shows (**a**) ROI (pink circle) in the area of maximum perfusion. (**b**) ROI (pink circle) in the area of minimum perfusion.
